# Preventive effects of regular physical exercise against cognitive decline and the risk of dementia with age advancement

**DOI:** 10.1186/s40798-015-0016-x

**Published:** 2015-04-17

**Authors:** Thierry Paillard

**Affiliations:** Département STAPS, Université de Pau et des Pays de l’Adour, ZA Bastillac Sud, 11 rue Morane Saulnier, 65000 Tarbes, France

## Abstract

With age advancement, cognitive function is impaired and the risk of dementia is increased under the influence of normal or pathological cortical and subcortical neuronal alterations. Significant researches has been undertaken to analyze the preventive effects of exercise against the decline of cognitive function and the risk of dementia (e.g., Alzheimer’s disease), particularly during the past 10 years. The aim of this short review is to report the scientific knowledge, relating to these effects, that has been obtained during the past 10 years. Acute physical exercise raises the cardiac output in response to increased needs for oxygen and energetic substrates compared to the state of rest, which increases the cerebral blood flow. The increased cerebral blood flow triggers various neurobiological mechanisms in the brain tissue. Repeated and regular physiological modifications related to exercise facilitate the synthesis of cerebral tissue. Regular physical exercise (rPE) may thus increase angiogenesis, neurogenesis, synaptogenesis, and the synthesis of neurotransmitters in different cerebral structures involved in cognition due to an increase in the liberation of neurotrophic factors and the production of enzymatic antioxidants. There is an inversely proportional relationship between the amount of physical activity undertaken and the risk of cognitive decline and/or the development of neurodegenerative disease. The synthesis of cerebral tissue under the influence of aerobic rPE may increase the volume of the gray and white matters of the prefrontal and temporal cortical areas as well as the volume of the hippocampus. Moreover, coordination exercise stimulates cognitive function, thereby inducing positive adaptations of cerebral function when regularly practiced. The possible effects of other types of exercise that weakly stimulate the cardiovascular system or cognitive function, such as stretching and strength training, are also beneficial but their mechanistic explanations require further exploration.

## Key points

- There is an inversely proportional relationship between the amount of physical activity undertaken and the risk of cognitive decline and dementia,
- Regular physical exercise may increase angiogenesis, neurogenesis, synaptogenesis in different cerebral structures involved in cognition, and
- Aerobic exercise activates and maintains the sensorimotor network while coordination exercise activates and maintains the visual-spatial network.

## Review

### Introduction

More than 50% of 85-year-old subjects present cognitive disorders ranging from simple non-pathological disorders of the memory to dementia states [1]. Cerebral alteration is inevitable as a result of age advancement even for clinically healthy subjects. The risk of dementia and the prevalence of neurodegenerative pathologies radically increase with age advancement [2,3]. Genetic and environmental factors also have an influence on the development of these pathologies [4-7]. In humans, the most frequent form of dementia is Alzheimer’s disease [8].

Moreover, indirect factors can have a significant negative influence on cerebral alteration with age advancement. These include social and familial factors (e.g., persons who are socially isolated), economic factors (e.g., persons with limited financial means), and available therapeutic means (e.g., lack of effective disease-modifying treatments) [9-12]**.** It is difficult for individuals to overcome these unfavorable factors. However, physical exercise constitutes an ecological approach (together with food and cognitive stimulation) likely to attenuate the effects of normal cerebral aging (non-pathological) and reduce the risk of dementia [13]. The physiological adaptations resulting from chronic exercise are predicted to be favorable to cerebral health.

Indeed, acute physical exercise raises the cardiac output in response to increased needs for oxygen and energetic substrates compared to the state of rest, which increases the cerebral blood flow [14]. The increased cerebral blood flow triggers various neurobiological mechanisms in the brain tissue. Repeated and regular neurobiological modifications related to exercise facilitate the synthesis of cerebral tissue by increasing angiogenesis, neurogenesis, synaptogenesis, and the synthesis of neurotransmitters in different cerebral structures involved in cognition (e.g., memorization) [15,16]. On the basis of the above data, regular physical exercise (rPE) is likely to maintain or even improve cognitive function of healthy elderly subjects [17] and reduce the risk of dementia - e.g., Alzheimer’s disease [18,19].

During the last decade, a number of observational studies as well as interventional studies have been carried out in order to determine the preventive effects of rPE against the decline of cognitive function and the risk of dementia. However, the question of the actual potential of rPE to generate neuroprotective mechanisms in healthy subjects who advance in age currently still remains open. The aim of this short review is to summarize the main scientific knowledge relating to the preventive effects of rPE against the decline of cognitive function and the risk of dementia (e.g., Alzheimer’s disease) that has been published in the past 10 years. Significant results obtained by empirical studies prior to this period are also included in this review as well as the mechanistic explanations of preventive effects induced by rPE.

An electronic search strategy was carried out using the *PubMed* database on the basis of reasonable expressions of these three concepts: physical activity (exercise, physical activity, physical exercise, physical training, motor activity), cognition (cognitive function, memory, executive function, cognitive impairment, cognitive decline, dementia, Alzheimer’s disease, brain), and age advancement (aging, older, elderly). A number of interventional and observational studies were identified as relevant and their methodology was in conformance with existing norms (number of subjects, protocol, etc.).

### Prevention of cognitive decline with age advancement

#### Normal cerebral aging in healthy subjects

With age advancement, memory, and cognitive decline in healthy subjects is partly explained, at least, by the degradation of the process of synaptic plasticity [20]. At the cellular level, one can observe a loss and/or a change of dendritic arborization accompanied by a disappearance and/or a modification of the synaptic structures [21]. The neurons undergo a slowing down of their metabolic activities; their average size decreases and the cerebral blood flow as well as the glucose metabolism decline [22,23]. In mammals, with age advancement, neuronal apoptosis particularly affects cerebral areas such as the neocortex, the hippocampus, and the cerebellum [24]. The neuronal losses especially concern the neurons whose axons are myelinated [25-27]. Moreover, intense chronic psychological stress and, more particularly, the concentration in glucocorticoids that it induces, aggravate the effects of cerebral aging. Prolonged exposure to high plasmatic concentrations of cortisol damages the neurons of the hippocampus [28,29]. Finally, normal cerebral aging generally engenders an alteration of dopaminergic pathways that are essential for the activity of the executive functions [30]. In healthy subjects, age advancement results in cerebral structural modifications which induce cognitive functional decline.

#### Preventive effects of regular physical exercise

Normal cerebral aging (non-pathological) can induce a cognitive decline which can be slightly to moderately limited by rPE. Observational studies have shown that rPE has a prophylactic effect on cerebral health and cognitive function of elderly people. A longitudinal study carried out in Western Europe (in Finland, Italy, and Holland) over a 10-year period revealed that subjects (*n* = 295) who decreased their level of daily physical activity in amount or in intensity showed a cognitive decline that was greater than those subjects who maintained their level of physical activity in amount or in intensity [31]. Moreover, in a cohort of 347 elderly men (74.6 ± 4.3 years), the cognitive decline measured by means of the mini-mental status examination (MMSE) test was higher for individuals who practiced less than 1 h of weekly physical activity than those who were significantly more active [32]. Lytle et al. [33] reported that 30 min of aerobic activity, three times a week during a period of 2 years (929 subjects who were 76 years old completed the whole study out of 1,146 subjects who were initially recruited) reduced cognitive decline by half (MMSE test), when compared with subjects who remained physically inactive. The risk of cognitive decline appears to be inversely proportional to the amount of physical activity practiced throughout life. A longitudinal study of 5,925 subjects aged more than 65 over a 6- to 8-year period revealed that the cognitive decline was 17%, 18%, 22%, and 24%, respectively, for the highest, the third, the second, and the lowest quartiles related to the amount of physical activity daily undertaken [34]. Another study of 18,766 American women, aged between 70 and 81, over a 7-year period (between 1995 and 2001 and between 1997 and 2003) indicated that the subjects belonging to the highest quintile in terms of level of physical activity presented a risk of cognitive decline 20% lower than the subjects belonging to the lowest quintile [35]. Moreover, the preventive effects of rPE on cognitive function are also evident in the case of very old subjects aged near to 80 [36].

#### Prevention of the risk of dementia

##### Physiopathological characteristics

In humans, the most frequent form of dementia is Alzheimer’s disease. It seems relevant to briefly describe the cerebral disorders of this neurodegenerative disease. Alzheimer’s disease is characterized by a loss of neurons and synapses in the cerebral cortex as well as in certain subcortical regions. The neuronal loss induces an atrophy of the affected regions such as the temporal lobe, the parietal lobe, and a part of the frontal cortex and the cingular gyrus [37]. Concerning the clinical aspects, the presence of amyloid plaques in the neocortex and the hippocampus reveals the existence of the disease and corresponds to the extracellular accumulation of Aβ-42 cerebral proteins which are neurotoxic. The presence of non-eliminated Tau (tubule-associated unit) proteins forming aggregates is also a sign of neurofibrillary degeneration (deterioration of the microtubules which form the cytoskeleton of axons) which prevents (blocks) the axonal transport of biological elements required for neuronal activity. Moreover, Alzheimer’s disease causes Aβ deposition in the cerebral vessel wall leading to cerebral amyloid angiopathy [38]. Aβ is particularly toxic to endothelial cells and inhibits endothelial nitric oxide synthase (eNOS) in cultured endothelial cells. Hypercontractility of the affected vessels, ultimately leading to chronic impairment of cerebral blood flow (cerebral hypoperfusion), thus contributes to neurodegeneration. Furthermore, vascular risk factors (hypotension, diabetes, hyperlipidaemia, elevated homocysteine, atrial fibrillation, etc.) are highly prevalent in old subjects and closely related to cognitive impairment in later life [38]. The neurological and cardiovascular factors are closely linked to the physiopathology of dementia (e.g., Alzheimer’s disease).

##### Preventive effects of regular physical exercise

The effects of rPE are actual and beneficial against the risk of dementia. There is a relationship between the practice of physical activity and Alzheimer’s disease. Larson et al. [39] have concluded through a longitudinal study that rPE delays the onset of Alzheimer’s disease and dementia in very old subjects. In humans, the results of epidemiological studies have also revealed other advantages. rPE may in fact limit or even prevent the development of Alzheimer’s disease, e.g., [40]. An American study of 1,740 subjects aged more than 65 showed that the incidence of dementia was 13.0 per 1,000 person-years for participants who exercised three or more times per week (≥15 min/session of walking, bicycle, swimming, aerobics, eurhythmics, aquaerobics, strength training, stretching, or other activities) compared with 19.7 per 1,000 person-years for those who exercised fewer than three times per week [39]. A meta-analysis has concluded that there is an inversely proportional relationship between the amount of rPE practiced and the risk of developing Alzheimer’s disease (0.55; 95% CI 0.36 to 0.84; *p* = 0.006) [13]. Other even more recent works have confirmed this relationship [18,19,41]. Karceski [42] specified, in a comparison of active subjects and inactive subjects, that the risk of developing the disease was twice as high for the inactive subjects. Hamer and Chida [13] deduced almost the same conclusion by showing that rPE reduced the risk of developing Alzheimer’s disease by 45%.

##### Neuroprotective mechanisms resulting from regular physical exercise

The repeated and regular physiological modifications (i.e., increased cerebral blood flow) related to exercise are favorable to the synthesis of cerebral tissue, e.g., [15,17]. rPE activates the neurotrophic function and angiogenesis, thereby facilitating neurogenesis and synaptogenesis which improve the memory and the cognitive functions, e.g., [15,43-45].

In order to analyze and describe the mechanisms involved in the protective effect of rPE, studies completed with animal models have been necessary. rPE carried out between moderate and high intensities has a neuroprotective effect by increasing the production of antioxidant enzymes (particularly superoxide dismutase or SOD), endothelial nitric oxide synthases (eNOS), brain-derived neurotrophic factors (BDNF), nerve growth factors (NGF), insulin-like growth factors (IGF-1), and vascular endothelial growth factors (VEGF), and by reducing the production of free radicals (reactive oxygen species or ROS) as well as the concentration of amyloid-ß plaques (Aβ), in particular in the cerebral zones involved in cognitive function (notably the memory) such as the hippocampus [38,46,47]. In a study carried out by Van Praag et al. [48] rPE induced hippocampic neurogenesis (particularly in the dentate gyrus) which was associated with synaptogenesis and an improvement in the learning capabilities (spatial memory and long-term potentiation) of trained old mice in comparison with control mice (same model and same age), that did not have access to a running wheel. Regular and continuous training of rats (from 5 to 23 months of age) over a period of 18 months, which required them to run on a horizontal treadmill at a speed of 20 m/min for 20 min, twice a day, 5 days a week, also had a neuroprotective effect on the cerebellum [49]. Larsen et al. [49] reported that sedentary-aged rats had 11% fewer Purkinje cells and 9% smaller Purkinje cell soma volumes (both two, *p* = 0.02) than exercised aged rats, and exercised aged rats had the same number of Purkinje cells as young rats (5 months of age). Evidence suggests that, in the case of animals, rPE results in beneficial structural cerebral adaptations.

In the case of cognitively normal human subjects, rPE is likely to improve the executive function, attentional capacity, processing speed, episodic memory, and procedural memory [36,50,51]. Physical exercise of 40 min repeated four times a week (ergocycle, treadmill, and stair-climbing) for a period of 12 weeks increased the cerebral blood flow in the dentate gyrus of the hippocampus which facilitates its neurogenesis [52]. rPE engendered an increase of gray matter in the temporal and frontal lobes which improved cognitive performances [17,53]. An aerobic program (three weekly sessions of 1 h for a period of 6 months) generated a volume increase (evaluation by means of magnetic resonance imaging or MRI) of gray and white matters in certain prefrontal and temporal cortical regions (i.e., those which are substantially damaged by age advancement) for subjects aged between 60 and 79 [54]. A longitudinal study showed that there is also a relationship between the amount of physical activity practiced and the volume of the prefrontal and temporal areas as well as the volume of the hippocampus, after 9 years of monitoring of 299 subjects (178 women) aged 78 [55]. This study specified that greater gray matter volume resulting from rPE reduced the risk for cognitive impairment. One year later, the same laboratory carried out an interventional study and reported that aerobic training over 12 months (3 days/week, 40 min/session, 60% to 75% of the maximum heart rate reserve from the seventh week) generated an increase (+2%) of the hippocampal volume (and it is known that the age-related hippocampal annual volume loss is between −1% and −2% a year) which was associated with an increase in the plasmatic concentration of BDNF in old healthy subjects (120 subjects randomized into two groups, 60 × 68-year-old control subjects versus 60 × 66-year-old experimental subjects who practiced stretching exercises) [56]. Evidence also suggests that, in the case of humans, aerobic exercise generates beneficial structural and functional cerebral adaptations.

Lange-Asschenfeldt and Kojda [38] have described the molecular mechanisms of the beneficial effects of rPE on the vasculature, such as activation of the vascular NO/eNOS pathway. These authors concluded that, as well as maintaining neuronal plasticity, rPE may prevent/counteract cerebral pathophysiology by building a vascular reserve. Moreover, in the case of cognitively normal subjects, rPE is associated with Alzheimer’s disease biomarkers. In a study of 61 adults aged between 55 and 88, confirmed to be cognitively normal, Liang et al. [57] recorded that individuals with elevated Pittsburgh compound-B or PIB (i.e., radiotracers were used to image amyloid-β aggregates with plaque formation, which indicated higher uptake of PIB) and cerebrospinal fluid Tau proteins and phosphorylated Tau proteins (ptau) carried out less exercise whereas active individuals had lower PIB binding. Nevertheless, the mechanical explanation is still uncertain since it has not yet been possible to attribute the causal link between the value of these biomarkers and the amount of physical activity.

rPE that focuses on motor coordination activates cerebral structures that are involved in executive control and perceptual speed, thereby improving not only the quality of motor responses (execution correctness and speed) but also the integration process of visuospatial information [58]. Voelcker-Rehage et al. [58] have shown with the use of the functional MRI technique that rPE that focuses on motor coordination enhances the activation level of the cerebral network that treats visuospatial information. These authors have specified that repeated and regular enhancements of the activation level induce structural and functional adaptations of this cerebral network thereby facilitating cognitive functioning.

For adults [17] and old adults aged between 60 and 79 [54], stretching and toning or relaxation interventions do not have any effect on the cerebral volume and cognitive function because the metabolic or cognitive demands are too low in comparison with aerobic exercise and coordination tasks. Aerobic exercise induces changes in the cardiovascular function that occur based on the energy demands, while coordination exercise induces changes in information processing that occur based on the cognitive demands. This effect of coordination exercise might be crucial for inducing molecular or cellular changes and thus might show the positive effects of physical activity on cognitive function [52,58]. Cardio-vascular training has been associated with increased activation of the sensorimotor network, whereas coordination training has been associated with increased activation of the visual-spatial network [58]. Nevertheless, other authors have reported that non-aerobic and non-visuo-motor coordination activities such as stretching and toning could improve the executive function in old subjects [59]. Tai Chi could also be beneficial for cognitive function [60-63] even if its effects need to be validated by studies of large cohorts of old subjects [64].

The contribution of each type of exercise to the cerebral structures and functions of old persons remains to be determined more precisely by future studies in this field. At this point, it is evident that aerobic exercise combined with other types of exercise (e.g., motor coordination, stretching, toning, Tai Chi) generates greater positive effects on cognitive function than aerobic exercise practiced alone [65].

## Conclusions

Regular aerobic exercise can limit cognitive decline and the risk of dementia as a result of various neurobiological mechanisms that it is likely to trigger in the brain tissue especially in the temporal and prefrontal areas as well as the hippocampus. This form of exercise can activate and maintain the sensorimotor network. However, the optimal workload related to the frequency, the intensity, and the duration of sessions has still not been clearly established, and future research on this topic should particularly focus on these aspects. Regular motor coordination exercise can also generate positive adaptations of cognitive function, and this form of exercise is likely to activate and maintain the visual-spatial network. The preventive effects of other types of exercise still need to be explored.
